# NFIXing Cancer: The Role of NFIX in Oxidative Stress Response and Cell Fate

**DOI:** 10.3390/ijms24054293

**Published:** 2023-02-21

**Authors:** Vanessa Ribeiro, Susana G. Martins, Ana Sofia Lopes, Sólveig Thorsteinsdóttir, Rita Zilhão, Ana Rita Carlos

**Affiliations:** 1 cE3c-CHANGE, Department of Animal Biology, Faculty of Sciences, University of Lisbon, 1749-016 Lisbon, Portugal; 2 Centro Hospitalar de Lisboa Ocidental (CHLO), 1449-005 Lisbon, Portugal; 3 cE3c-CHANGE, Department of Plant Biology, Faculty of Sciences, University of Lisbon, 1749-016 Lisbon, Portugal

**Keywords:** NFIX, oxidative stress, cell fate, cancer, development

## Abstract

NFIX, a member of the nuclear factor I (NFI) family of transcription factors, is known to be involved in muscle and central nervous system embryonic development. However, its expression in adults is limited. Similar to other developmental transcription factors, NFIX has been found to be altered in tumors, often promoting pro-tumorigenic functions, such as leading to proliferation, differentiation, and migration. However, some studies suggest that NFIX can also have a tumor suppressor role, indicating a complex and cancer-type dependent role of NFIX. This complexity may be linked to the multiple processes at play in regulating NFIX, which include transcriptional, post-transcriptional, and post-translational processes. Moreover, other features of NFIX, including its ability to interact with different NFI members to form homodimers or heterodimers, therefore allowing the transcription of different target genes, and its ability to sense oxidative stress, can also modulate its function. In this review, we examine different aspects of NFIX regulation, first in development and then in cancer, highlighting the important role of NFIX in oxidative stress and cell fate regulation in tumors. Moreover, we propose different mechanisms through which oxidative stress regulates NFIX transcription and function, underlining NFIX as a key factor for tumorigenesis.

## 1. Introduction

The nuclear factor I (NFI) family of transcription factors controls the expression of several genes that play a role in various cellular processes (e.g., proliferation, migration, and differentiation) during normal development, as well as in the context of disease, including cancer [1,2]. This family includes four closely related genes, *NFIA*, *NFIB, NFIC,* and *NFIX*, present in human chromosomes 1p31.2-p31.3 (*NFIA*), 9p24.1 (*NFIB*) and 19p13.3 (*NFIX*, *NFIC*), whose proteins share a highly conserved N-terminal DNA-binding and a dimerization domain [3]. The binding of NFI proteins to DNA occurs in the form of homodimers or heterodimers, which increases the diversity of targets for these transcriptional regulators [4]. Additionally, a recent study on the interaction between a large universe of transcription factors showed that 118 out of the 202 interactions analyzed involved members of the NFI family [5]. This suggests that NFI family members may play a variety of roles in the regulation of transcription, either acting directly as activators or repressors or by interacting with other proteins, namely transcription factors, to modulate their function [1].

While NFI family members share several common features, allowing for compensatory roles [6,7], they also have specific regulatory functions [8,9]. Important information about these specific roles comes from the analysis of knockout mice for different NFI family members [10]. For example, NFIA, NFIB, and NFIX play an important role in glial and neuronal differentiation in the central nervous system [10], while NFIC plays a specific role in tooth development [8], and only NFIX plays a role in muscle development [9]. Not surprisingly, alterations in the expression of these genes can lead to several pathologies, including developmental defects and cancer [2,10]. This multi-faceted family of transcription factors has also been implicated in the regulation of epigenetic modifications in various ways, possibly due to their transactivation domain that interacts with histones H1 and H3 or through the binding and modulation of the activity of different chromatin modifiers [11,12,13]. The global effect of NFI-chromatin interactions seems to be the increase in chromatin accessibility and gene expression [14,15]. Likewise, NFI proteins were shown to positively regulate transcription by recruiting histone acetylases and nucleosome remodeling enzymes (e.g., NURF) and to drive an increase in active chromatin modifications, such as H3K4me3 and H3K36me3 [16].

Apart from the role that NFI transcription factors play in regulating gene expression, they are themselves regulated at different levels. This regulation may occur at (i) the transcriptional level, (ii) the post-transcriptional level via alternative splicing, (iii) the mRNA stability and translational level, regulated by different non-coding RNAs, and (iv) the post-translational level [1,10,17,18,19]. Transcriptional regulation may occur, for example, through the action of paired box gene 6 (PAX6) or empty spiracles homolog 2 (EMX2), two transcription factors that allow the transcription of NFI family members [1,17,18]. NFI family regulation by non-coding RNAs has been addressed particularly in the context of cancer and includes: (i) microRNAs (miRNAs), which are 18–25 nucleotide long abundant non-coding RNAs that inhibit translation or promote degradation of messenger RNAs (mRNAs) and can stimulate proliferation and migration [20,21,22,23,24,25]; (ii) circular RNA (circRNA), which are non-coding RNAs, most of them originated from protein-coding exons that sponge and regulate the activity of miRNAs or serve as protein decoys to recruit and modulate the transcription and translation of downstream target genes [26,27,28]; and (iii) long non-coding RNAs (lncRNAs), which are functional 200 nucleotide transcripts that mainly modulate transcription through a variety of epigenetic mechanisms, post-transcriptional processing via cross-talk with other RNA species, or modulate gene expression through lncRNA-protein interactions [29,30]. Finally, an example of a post-translational modification of the NFI family transcription factors is the conserved cysteine residue that has been shown to undergo oxidative inactivation [19]. This oxidative inactivation of NFI transcription factors, proposed as important for cellular responses to oxidative stress, can be reverted by the glutathione antioxidant pathway [31]. Although more and more examples of this multilevel regulation of NFI transcription factors are being discovered, the choice of downstream target genes and pathways, which are decisive for developmental and disease processes, still needs to be clarified.

Cancer is a complex disease characterized by multiple events known as hallmarks [32,33]. These hallmarks are associated with a profound change in the cell’s expression profile, allowing cancer cells to acquire the ability to proliferate, migrate and regain certain characteristics of stem cells. The increased plasticity of cancer cells often correlates with a blockage in differentiation, which, in its turn, depends on alterations in the expression of transcription factors that play key roles during development, such as the HOX, SOX, and PAX families [32]. The co-option of developmental pathways during cancer onset and progression has been described [32,34,35,36], raising the possibility that NFI proteins may play a relevant role in cancer [2,7,10,11,17].

In this review, we build on the existing knowledge about the role of NFIX during development to examine its role in cancer. Thereafter, in the context of cancer, we will focus on how NFIX relates to oxidative stress and alters cell fate and how that impacts tumor progression.

## 2. NFIX Roles in Development

**Figure 1:**
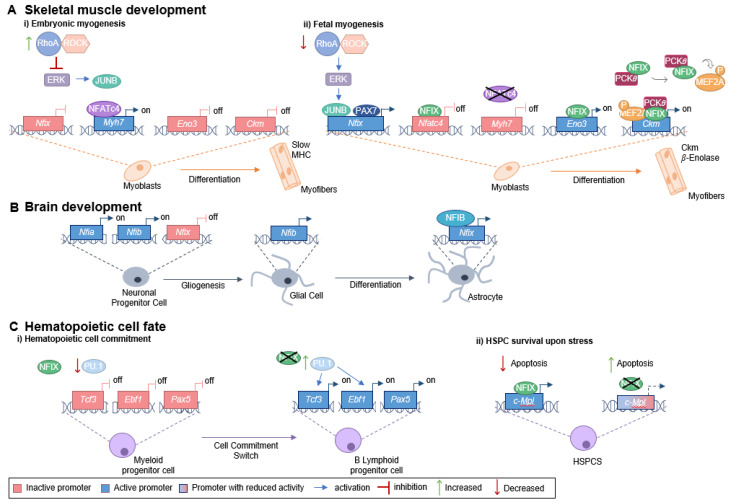
**Role of NFIX in development**. (**A**) NFIX promotes the switch between embryonic and fetal myogenesis: (i) During embryonic development, embryonic muscle-specific genes are expressed, including *Myh7* (encoding slow MHC), which is activated by NFATc4 that binds to the *Myh7* promoter. The RhoA/ROCK axis promotes the embryonic identity of myoblasts through the repression of ERK kinases, JUNB and NFIX. (ii) In the transition from embryonic to fetal muscle development, RhoA/ROCK activity decreases, which leads to increased ERK activity and subsequent activation of JUNB. The transcription factor PAX7 also binds to the *Nfix* promoter activating its transcription. NFIX binds to the *Nfatc4* promotor inhibiting *Nfatc4* expression and, consequently, slow MHC is not produced. On the other hand, NFIX activates fetal-specific genes, such as *Ckm* and *Eno3* (which encodes β-enolase)*,* its downstream targets. NFIX binds directly to the *Eno3* promoter, activating its transcription, while activation of the *Ckm* promoter involves a MEF2A/NFIX/PKC𝜃 complex. (**B**) Glial cell differentiation is promoted by NFIX: the expression of *Nfia* and *Nfib* by neuronal progenitor cells leads to a gliogenic switch. Then, NFIB binds the *Nfix* promoter region activating its transcription, and, in its turn, NFIX activates the astrocytic genes leading to astrocyte differentiation. (**C**) NFIX regulates hematopoietic cell fate: (i) During a stressful event, NFIX activates the *c-Mpl* promoter directly, leading to a reduction in the apoptosis of hematopoietic stem and progenitor cells. (ii) NFIX also prevents early B cell development and favors myeloid differentiation. PU.1 regulates the transcription factors E2A (encoded by *Tcf3*), EBF (encoded by *Ebf1*), and PAX5. In the presence of NFIX, PU.1 levels are lower, and therefore, the expression of *Tcf3*, *Ebf1*, and *Pax5* decreases, favoring myeloid fate. When NFIX is absent, the expression of PU.1 increases, enabling the activation of key genes required for B cell lymphoid lineage commitment (*Tcf3*, *Ebf1*, and *Pax5*).

To understand the role being played by NFIX in cancer, it is important to characterize its function during development. During mouse fetal myogenesis, NFIX activates specific fetal muscle genes, such as those encoding enolase-β (*Eno3*) and muscle creatine kinase (*Ckm*), both known downstream targets of the NFIX pathway [9] (Figure 1A). The transcription factor PAX7 (a key muscle stem cell marker) binds to the *Nfix* promotor to activate its expression. *Nfix* transcription also occurs through the action of JUNB (a member of the AP-1 family of transcription factors), which binds to the *Nfix* promotor via an unknown mechanism [37]. JUNB is activated downstream of ERK kinase signaling, which is low during embryonic myogenesis but increases at the beginning of fetal myogenesis due to a decrease in the RhoA/ROCK axis [37]. The activation of NFIX is, thus, downstream of a switch between RhoA/ROCK and ERK signaling, which occurs precisely at the onset of fetal myogenesis (Figure 1A) [37]. NFIX, in its turn, activates the expression of the fetal muscle-specific genes and inhibits the transcription of embryonic muscle-specific genes, such as slow myosin heavy chain (slow *MHC*, encoded by *Myh7*), marking the transition between embryonic and fetal muscle development [9].

NFIX is normally not expressed in adult muscle stem cells (satellite cells), but its abnormal activation is associated with disease progression, namely in the context of muscular dystrophies [38]. Muscular dystrophies are a group of diseases characterized by loss of muscle mass, fibrosis, and chronic inflammation, which are frequently associated with an increase in oxidative stress [39,40,41,42]. The deleterious role played by NFIX in the context of muscular dystrophies has been associated with consecutive cycles of regeneration and degeneration [38]. Additionally, NFIX is thought to contribute to increased oxidative stress both by driving regeneration in dystrophic muscles and by countering the switch of myofibers towards oxidative slow-twitch fibers, which are thought to reduce oxidative stress [9,38,43]. Consequently, this supports the idea that NFIX has a role in regulating oxidative stress levels in the muscle. During muscle regeneration, there is a close interaction between myogenic cells and macrophages, where NFIX regulates macrophage differentiation [44]. Under this scenario, injury-activated satellite cells attract blood monocytes that infiltrate into the damaged muscle and differentiate into pro-inflammatory macrophages, which, in their turn, stimulate myoblast proliferation [44]. Macrophages then switch to an anti-inflammatory phenotype, which sustains myogenic differentiation [45]. This phenotypic change is controlled by NFIX, which becomes activated in response to the inhibition of the RhoA/ROCK axis and by the induction of phagocytosis, a necessary feature for the acquisition of anti-inflammatory phenotype [44]. The anti-inflammatory phenotype enhances tissue repair and promotes fibroblast proliferation, which may lead to fibrosis and is highly detrimental to dystrophic muscles [44,46,47,48,49,50].

In addition to the crucial role in skeletal muscle development, *Nfix* is also expressed within the nervous system throughout embryogenesis [51]. In mouse cortical development, NFIX promotes the timely generation of intermediate progenitor cells that will originate cortical neurons through the transcriptional activation of the *Insc* (encoding inscuteable protein) [52]. Moreover, during mouse spinal cord development, the transition from producing neurons to producing glial cells (gliogenic switch) occurs via sequential action of NFI transcription factors [53]. In particular, during this gliogenic switch, NFIX has been shown to act downstream of NFIA and NFIB [53] (Figure 1B). NFIX has also been described to promote the differentiation of neural progenitor cells within the developing neocortex and hippocampus, triggering cell cycle exit via the transcriptional repression of SOX9 [54], a transcription factor required for the self-renewal of cortical neural progenitors [55]. Additionally, NFIX is an important regulator of proliferation and migration in the subventricular zone of the neurogenic niche during mouse embryonic development, a region that continuously generates neurons throughout adult life [54]. NFIX also plays a key role postnatally, by maintaining proliferative progenitor cells in this region, such as those expressing *Pax6*, *Sox2*, *Hes1* and *Hes5*, and *Mash1*, markers for progenitor or transit-amplifying cells [54]. The regulation of proliferation and migration, mediated by NFIX, may be associated with the neuroblast chemoattractant GDNF (glial cell-derived neurotrophic factor), whose gene has been shown to be a potential target for transcriptional activation by NFIX [56]. In addition, NFIX has been implicated in the regulation of post-mitotic cell migration within the hippocampus [54] and rostral migratory stream [56].

NFIX is also involved in the proliferation and repopulation activity of hematopoietic stem and progenitor cells [2]. This transcription factor has been implicated as a modulator of hematopoietic cell fate since its expression prevents early B cell development and favors myeloid differentiation [57]. The transcription factor PU.1, known to control myeloid and early B and T-cell development, and the transcription factors E2A (encoded by *Tcf3*), EBF (encoded by *Ebf1*), and PAX5 [58], necessary for B cell lineage commitment and development into mature B cells, are altered in the presence of NFIX [57] (Figure 1C). Moreover, during a stressful event, such as a hematopoietic stem and progenitor cells transplant, NFIX regulates c-MPL (thrombopoietin receptor or myeloproliferative leukemia protein) signaling pathway, promoting the survival of the hematopoietic stem cells [59]. It does so by directly activating the c-MPL promoter, which regulates the maintenance of hematopoietic stem cells in the bone marrow niche, promotes their survival via JAK/STAT and MAPK/ERK signaling cascades, and prevents apoptosis (Figure 1C) [59].

NFIX has also been shown to play a key role in meiosis during spermatogenesis, with NFIX deficiency leading to a blockage in prophase 1 (diplotene), possibly associated with a defect in the synaptonemal complex and accumulation of DNA damage in mouse spermatocytes [60]. The possible role of NFIX as a cell cycle checkpoint regulator during human spermatogenesis was further suggested by another study where the regulation of NFIX expression during spermatogenesis was shown to be controlled by the microRNA miR-663 [23]. Silencing NFIX stimulated proliferation, possibly by increasing the expression of Cyclin A2, Cyclin B1, and Cyclin E1, and DNA synthesis, and inhibited apoptosis of human spermatogonia stem cells [23].

NFIX may also play a role in heart development, even though the data available is still scarce [61]. Interestingly, circRNAs have been indicated in several studies as playing a key role in physiological processes in various diseases, including the initiation and progression of cardiovascular diseases [62,63]. One such example is circNFIX (a circRNA derived from NFIX), which has been suggested to play a role in cardiac development and disease [64,65,66]. Moreover, the downregulation of circNFIX has been shown to lead to increased cardiomyocyte proliferation and angiogenesis [65], supporting the idea that circNFIX downregulation could be important for cardiac regeneration after injury. In addition, circNFIX counters heart hypertrophy by indirectly targeting activating transcription factor 3 (ATF3) in cardiomyocytes through binding to the microRNA miR-145-5p [64]. ATF3, a member of the cAMP response element-binding protein/ATF family, has been linked to heart hypertrophy [67,68]. Research has shown that, by regulating the miR-145-5p/ATF3 axis, circNFIX can attenuate pressure overload-induced cardiac hypertrophy [64]. Additionally, circNFIX expression is altered in response to increased levels of oxidative stress [28]. For example, in the fetal cardiomyocyte-derived H9c2 cell line, circNFIX was found to be downregulated after treatment with the pro-oxidant agent hydrogen peroxide, which correlated with reduced apoptosis [28]. Furthermore, overexpression of circNFIX promoted apoptosis in this model, possibly by reducing the cellular response to oxidative stress [28]. These results suggest that regulation of circNFIX or NFIX may impact heart development and disease through a mechanism that is linked to the oxidative stress response. The crosstalk between NFIX and oxidative stress extends beyond the heart. For example, its expression contributes to increased oxidative stress in response to optic nerve crush in the retina [69].

Taken altogether, these studies allow us to conclude that NFIX plays multiple roles during the development of a variety of tissues, influencing cell proliferation, cell fate, and differentiation. It also affects cell migration, apoptosis, and oxidative stress. These cellular processes are either well-documented hallmarks or emerging hallmarks of cancer [32,33], opening the possibility of an important role of NFIX in cancer.

## 3. Roles of NFIX in Cancer

Tumorigenesis is characterized by the gain of malignant properties, including sustained proliferative signaling, phenotypic plasticity, and epigenetic reprogramming, all features also observed during embryonic development [32]. Not surprisingly, several pathways that play central roles during development are also altered during tumorigenesis. This is the case of RhoA/ROCK and JUNB signaling pathways that regulate NFIX expression during myogenesis and are involved in cancer cell proliferation and invasion [70,71]. In prostate cancer cells, SOX4, a transcription factor involved in the development of various tissues and which is commonly overexpressed in tumors [72], is overexpressed and activates NFIX [73]. Moreover, the overexpression of acyl-CoA synthetase 4 in the MCF-7 breast cancer cell line leads to changes in various developmental pathways, including the overactivation of *NFIX* and its target gene *ENO3* [74].

Apart from its positive and negative transcriptional regulation, genomic analysis of the *NFIX* gene in various tumors has revealed several mutations, including gene fusions [75]. Gene fusions are chromosomal rearrangements, usually involving insertions, deletions, inversions, or translocations, where two independent genes fuse together to form a hybrid gene [76]. These fusions have been studied primarily in the context of hematological and mesenchymal malignancies, but they also contribute to epithelial tumors [76]. Even though the role of *NFIX* in gene fusions is still not fully understood, it is likely that most of the gene fusions involving this gene have oncogenic properties (Table 1). This is the case of *NFIX-MAST1* [77] fusions in breast cancer and may also include the *NFIX–PKN1* translocation, described in carcinoma of the skin [78], the *BSG-NFIX* fusion identified in breast cancer [79] and the *NFIX–STAT6* gene fusion, which was identified in a tumor lesion with histological features of a solitary fibrous tumor [75].

To understand the role of NFIX in cancer, it is essential to know how the gene fusions, epigenetic changes, non-coding RNAs targets, and mutations in *NFIX* and in its regulatory elements contribute to specific pathways that drive tumor progression. This can reveal when *NFIX* acts as an oncogene and when it acts as a tumor suppressor (Table 1).

### 3.1. NFIX and Oxidative Stress

Tumors are characterized by increased levels of oxidative stress, which impact tumorigenesis in different ways, including by (i) triggering DNA damage; (ii) altering signaling pathways involved in cell proliferation and tumor growth; (iii) leading to chronic inflammation in the tumor environment; and (iv) changing the composition of the extracellular matrix, which impacts cell survival, proliferation, migration, and adhesion [41,83,84,85,86].

Members of the NFI family are thought to be pro-oxidants, and their inactivation is crucial for proper oxidative stress response [19]. NFIX may act as an oxidative stress producer, for example, by activating the transcription of *CYP1A1* (encoding cytochrome P450 1A1), which has an NFI binding site in the promoter region [87]. CYP1A1 is known to be pro-carcinogenic [88] and, similarly to other monooxygenases, leads to the generation of reactive oxygen species (ROS) as part of its catalytic activity [89,90]. Under normal conditions, the expression of CYP1A1 is suppressed, possibly due to an autoregulatory loop that controls the expression of CYP1A1 via CYP1A1-based hydrogen peroxide production and the NFI family [89,91]. However, when deregulated, the increased production of ROS and the production of pro-oncogenic metabolites may contribute to tumor progression [88]. Studies have shown that *CYP1A1* is upregulated in breast [91], bladder [92], and colon cancers [92]. Accordingly, the knockdown of *CYP1A1* has been found to downregulate ERK and PI3K/AKT pathways and to induce the AMPK pathway, leading to a reduction in tumor progression and cancer cell survival [91]. Supporting the idea that oxidative stress impacts the function of NFI family members, hepatoma cell lines treated with the pro-oxidant hydrogen peroxide or L-buthionine- (S,R)-sulfoximine showed impaired NFI binding to its DNA binding site due to increased oxidative stress, resulting in the inhibition of its function as a transcription factor [87].

Analysis of oxidative stress-related differentially expressed genes using data from 594 lung adenocarcinoma patients revealed that *NFIX* is downregulated in this type of cancer and has a direct correlation with poor prognosis [93]. This study proposed that *NFIX* downregulation serves as a mechanism for cancer cells to reduce ROS production, thus, increasing their fitness [93]. Similarly, another study found that NFIX upregulation is associated with poor prognosis in breast cancer because of its role in ROS status [94]. This indicates that *NFIX* may be used as a key gene in a ROS scoring system to predict prognosis and therapeutic efficiency. NFIX has also been identified as part of the common mitochondrial defect signature genes in hepatocellular carcinoma, which are genes activated in response to mitochondrial dysfunction, a major source of ROS in organisms [95], and associated with poor prognosis and reduced overall survival [96].

Besides NFIX protein being associated with oxidative stress in different contexts, circNFIX has also been shown to have an impact on both tumor progression and oxidative stress [28,97,98]. For example, circNFIX was found to promote cancer progression by upregulating glycolysis, as well as glucose uptake in glioma [99] and in non-small cell lung cancer [100], which can lead to overproduction of ROS in the context of diabetes [101]. In glioma, tumor progression was associated with the suppression of miR-378e and consequent expression of ribophorin-II (*RPN2*) [99], a target of miR-378e that promotes increased ROS and glycolysis [99,102]. Similarly, in non-small cell lung cancer, tumor progression was associated with the suppression of miR-212-3p and upregulation of ADAM10 [100], a protein that has been shown to be involved in oxidative stress-related conditions, such as cancer, Alzheimer, neurodegeneration, and inflammation [103]. Further research is needed in order to understand whether NFIX’s role as a pro-oxidant contributes to ROS accumulation in tumors and therefore promotes genomic instability, increased proliferation, and differentiation. In support of this notion, studies are recognizing NFIX and its target genes/proteins that are involved in oxidative stress as potential therapeutic targets for cancer therapy [91,93,94,99].

### 3.2. NFIX and Cell Fate

Given the pleiotropic role of NFIX during development, it is not surprising that changes in *NFIX* expression can significantly influence proliferation and differentiation. Apart from NFIX’s indirect role in proliferation through its involvement in oxidative stress, NFIX has also been shown to be involved in cell cycle regulation and cell fate decisions, which are closely linked to proliferation. For example, *NFIX* downregulation has been shown to reduce proliferation and cell viability in lung cancer [80] but to lead to increased proliferation in the context of endometrial carcinoma [21] and colorectal cancer [20]. On the other hand, overexpression of NFIX in esophageal squamous cell carcinoma has been shown to reduce cell proliferation and induce cell cycle arrest in G1/G0 phase [25].

**Figure 2:**
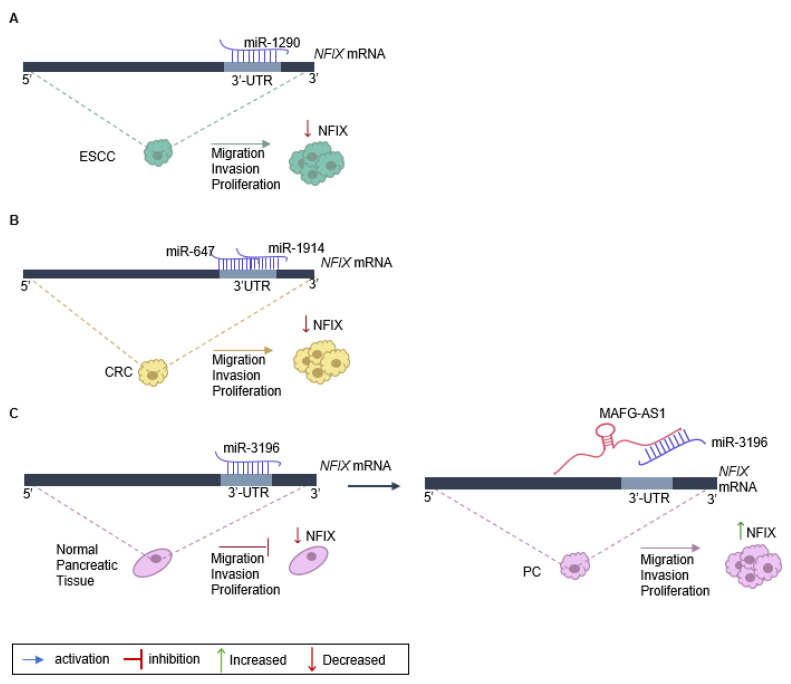
**Regulation of NFIX expression in cancer**. (**A**) *NFIX* regulation by miR-1290 promotes esophageal squamous cell carcinoma (ESCC) progression: miR-1290 directly targets the 3′UTR sites of *NFIX* mRNA, negatively regulating its expression. The decrease in *NFIX* expression leads to ESCC cell proliferation, migration, and invasion. (**B**) *NFIX* co-regulation by miR-647 and miR-1914 in colorectal cancer (CRC): *NFIX* mRNA is co-targeted by miR-647 and miR-1914 in the 3′UTR. The negative regulation of NFIX expression leads to CRC cell migration and invasion. (**C**) ceRNA network of MAFG-AS1/miR-3196/NFIX in pancreatic cancer (PC): in normal pancreatic tissue, miR-3196 directly binds to 3′UTR sites of *NFIX* mRNA and silences its expression. The lncRNA MAFG-AS1, highly expressed in PC cells, binds directly to the miR-3196, promoting *NFIX* upregulation and, as a consequence leading to proliferation, migration, and invasion of PC cells.

The role of NFIX in cancer proliferation, migration, and invasion has been linked to the expression of non-coding RNAs, namely miRNA and lncRNA (Table 1). One example is the regulation of NFIX mediated by miR-1290, which has a target site on the *NFIX* 3′-UTR [25] (Figure 2A). An inverse correlation between the levels of miR-1290 and NFIX protein and mRNA was observed in esophageal squamous cell carcinoma tissue samples, suggesting that miR-1290 is an oncogene that downregulates *NFIX* and promotes proliferation, migration, and invasion in this type of tumor [25]. Moreover, analysis of the genetic profile of colorectal cancer tissue through screening of genes that were upregulated or downregulated identified increased expression of two miRNAs, miR-1914 and miR-647, in colorectal cancer specimens and cell lines [20]. These miRNAs were shown to promote the proliferation and migration of colorectal cancer cells, functioning as oncogenes, possibly by directly targeting and downregulating NFIX (Figure 2B).

The impact of NFIX on proliferation has also been associated with lncRNAs that play diverse roles in regulating gene expression [104]. Numerous lncRNAs can act as competing endogenous RNAs (ceRNAs) to regulate the expression of coding genes that have common miRNA response elements [105], with pancreatic cancer being one example. In normal pancreatic tissue, miRNA-3196 is expressed, leading to a downregulation of NFIX [30]. However, in pancreatic cancer tissue, the lncRNA MAFG-AS1 acts as a ceRNA and binds to the miR-3196, resulting in the neutralization of miR-3196 and the upregulation of NFIX [30]. Functional assays have shown that MAFG-AS1 knockdown suppresses cell proliferation and migration while promoting cell apoptosis in pancreatic cancer [30]. Additionally, when miR-3196 is up-regulated, the proliferative and migratory capacities of pancreatic cancer cells are inhibited (Figure 2C).

In addition to cell cycle regulation and cell proliferation, NFIX may also play a role in other cell fates. Apoptosis is a central pathway that is rendered inactive in cancer cells [22,59,106]. It was recently shown that NFIX overactivation has an anti-apoptotic effect via the STAT5 signaling pathway leading to a reduction in apoptosis levels in hematopoietic stem and progenitor cells [59]. This is supported by the observation that the overactivation of NFIX leads to increased expression of the anti-apoptotic factor *Bcl2l1* (encoding BCL-XL) in these cells [59]. Additionally, *NFIX* downregulation through overexpression of miR-744-5p in ovarian cancer has been shown to decrease the expression of BCL2, an anti-apoptotic factor, leading to an increase in apoptosis levels [22]. Moreover, hematopoietic stem and progenitor cells that lack NFIX cannot survive in the bone marrow after transplantation due to an increase in apoptosis [107]. Nevertheless, *NFIX* silencing in the context of human spermatogonia stem cells seems to suppress early apoptosis [23], suggesting that its role in apoptosis may be tissue and/or cell-type-dependent.

Considering the important role of the NFI family in neuronal development, several studies have analyzed NFIX’s role in glioblastomas as a potential tumor-promoter [81,107,108]. One such study found that NFIX promotes glioblastoma cell migration by directly upregulating the expression of *EZR* (encoding ezrin), which is involved in linking the actin cytoskeleton and the plasma membrane and plays a role in cell migration [81] (Table 1). In accordance with the role of NFIX promoting cell migration, NFIX has been identified as a potential oncogene that plays a role in the development of metastasis. NFIX was recently described as a master regulator activating the expression of 17 genes that are involved in migration and invasion in lung cancer [80]. Using two different cell lines for lung cancer, it was shown that NFIX regulates interleukin-6 receptor subunit β (IL6ST), metalloproteinase inhibitor 1 (TIMP1), and integrin β-1 (ITGB1) genes, all of which are involved in cell proliferation, migration, and invasion [80] (Table 1). Altogether these studies suggest that NFIX may be a key player during cancer onset and progression, modulating several pathways implicated in tumorigenesis.

## 4. Discussion

**Figure 3:**
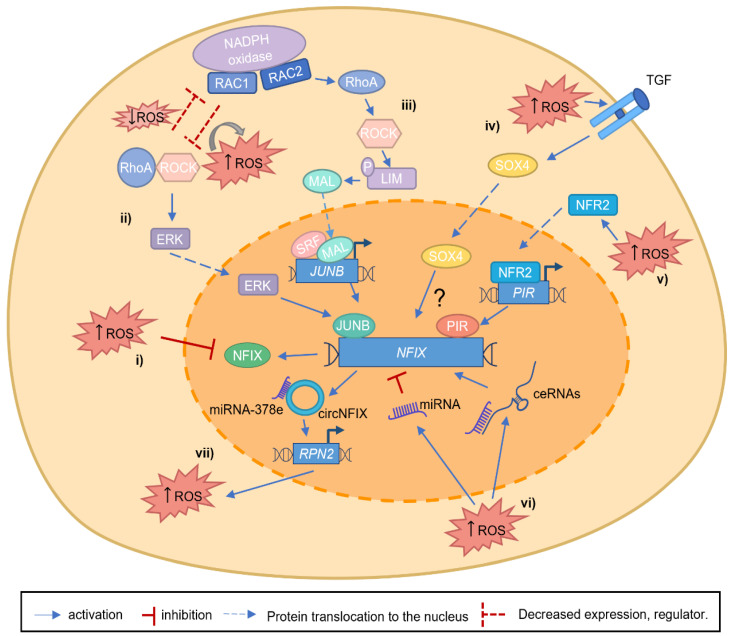
**Putative mechanisms linking NFIX and increased ROS levels**. (**i**) During tissue development, ROS can regulate NFIX expression, oxidizing specific NFIX cysteine residues. (**ii**). RAC1, a catalytic subunit of NADPH oxidase, can generate ROS and interact with RhoA/ROCK axis leading to its activation or inhibition. On the other hand, RhoA, through its target gene ROCK, can inhibit RAC1 activity, decreasing ROS levels. When RAC1 is able to activate the RhoA/ROCK axis and increase ROS levels, ERK kinases are activated, leading to JUNB activation, and consequently, JUNB activates *NFIX* expression. (**iii**) The increased expression of *RAC2,* and subsequent ROS production, lead to the activation and increase in RhoA and its targets, such as ROCK, that will phosphorylate LIM kinase. With LIM kinase phosphorylation, MAL translocates to the nucleus activating SRF. Consequently, SRF activates *JUNB* expression, and JUNB protein binds to the *NFIX* promoter, activating its transcription. (**iv**) NFIX is a putative target gene of SOX4, but the exact mechanism remains unknown (question mark). *SOX4* activation is mediated by ROS/TGFβ, and it is possible that this protein can translocate to the nucleus activating *NFIX* expression. (**v**). The oxidative stress sensor NRF2 is activated by increased ROS levels, allowing its translocation to the nucleus, where it activates the Pirin (*PIR*) promoter. Consequently, *NFIX* expression is induced through the binding of Pirin to the NFI I/CCAAT box transcription factor (NFI/CTF1) domain. (**vi**) ROS can lead to the activation of miRNAs or ceRNAs (network composed by miRNA and the inhibitory binding through lncRNA), which lead to a decrease or an increase in *NFIX* expression, respectively. (**vii**) In gliomas, miR-378e functionally targets RPN2, an oncogene, and ROS inducer, inhibiting its oncogenic functions. circNFIX can sponge the action of miR-378e, allowing RPN2 activity (as part of the ceRNA network), contributing to glioma progression.

NFIX has been established as a central transcription factor during development, for example, by promoting the switch between embryonic and fetal myogenesis [9] and in adulthood, being required for muscle regeneration [38,43]. The role of NFIX in mediating the switch between different cellular differentiation stages is not unique to muscle. For example, it occurs during the production of glial cells [53] or during hematopoietic cell fate [57]. This raises the possibility that NFIX is a critical factor for cell differentiation, which may be critical during tumor progression and metastasis. While some studies have suggested that NFIX may have a putative role as a tumor suppressor, most studies have identified NFIX as an oncogene (Table 1). However, the exact mechanisms that contribute to the alterations of *NFIX* mRNA or protein expression in cancer have not yet been fully described. One possible mechanism explaining the importance of NFIX in cancer might be dedifferentiation, which has recently been proposed as an emerging cancer hallmark [32]. In this scenario, it is possible that the control of *NFIX* expression leads to changes in the differentiation status and promotes a stem cell-like phenotype in cancer cells. This is in line with the role of NFIX in development, where it has been shown to control the differentiation stage of various cell types, including muscle [9,38], nervous system [54], and hematopoietic lineages [57,106,109]. The mechanisms controlling NFIX are diverse, and it is possible that several of these mechanisms may be regulated, or be regulated by, the production of ROS. Apart from the direct regulation of NFIX by ROS through the oxidative inactivation of its cysteine residues [19,31] (Figure 3i), other pathways that control NFIX activation may also be modulated by oxidative stress. For example, RhoA/ROCK and JUNB signaling pathways [70,71] or SOX4 overexpression [73], which control *NFIX* expression both during embryonic development and in the context of cancer, have been linked to oxidative stress. RhoA/ROCK pathway has been shown to have a bi-directional inhibitory effect of the RAC GTPase RAC1, which leads to ROS generation via NADPH oxidase [110,111,112] (Figure 3ii). This can occur during the phagocytosis of pathogens and apoptotic cells by macrophages, where ROS production may control *NFIX* expression [110,111,112]. Another RAC GTPase and catalytic subunit of NADPH oxidase, RAC2, has also been described to promote transcriptional activation of *JUNB* in lung cancer [71] (Figure 3iii), which could be yet another mechanism that leads to *NFIX* activation. In the scenario of high levels of ROS produced via NADPH oxidase, RhoA becomes activated and subsequently leads to the activation of the downstream targets, such as ROCK. This then allows ROCK to phosphorylate LIM kinase, leading to F-actin stabilization. With LIM kinase upregulation, MAL (megakaryocytic acute leukemia) can no longer be sequestered by actin monomers and translocates to the nucleus, where it activates SRF (serum response factor), a factor that responds to morphological changes in the actin cytoskeleton. RAC GTPases are well known for their role in cancer progression [110,113]. It is possible that NFIX is one of the downstream targets promoting tumorigenesis through increased proliferation, migration, and metastasis. SOX4 was shown to be activated via TGF-β and ROS, promoting cell senescence [114] (Figure 3iv). Another piece of evidence supporting the important role of NFIX in oxidative stress regulation is the fact that NFI I/CCAAT box transcription factor (NFI/CTF1) domain, present in the NFIX family members, was shown to interact with pirin [115] (Figure 3v). Pirin is an iron-binding protein, which is involved in iron metabolism, one of the sources of oxidative stress in organisms, and is regulated by NRF2 (nuclear factor erythroid 2-related factor 2), a major regulator of antioxidant response [116,117]. The role of pirin in cancer has been widely studied in the last decades [118]. Pirin is overexpressed in various types of cancer, such as colorectal cancer [116] and melanoma [119]. Therefore, it is possible that oxidative stress-dependent pirin activation allows its interaction with NFIX, modulating the activation of its downstream target genes. In addition, the important role that miRNA and lncRNA play in regulating *NFIX* expression may also be linked to oxidative stress (Figure 3vi). There is a growing body of evidence showing that miRNA and lncRNA lead to either a pro-oxidant or antioxidant response [120,121,122,123,124]. This is the case of miR-212-3p, which contributes to oxidative stress [125,126]. Finally, another link between NFIX and oxidative stress comes from circNFIX [64,65,66] (Figure 3vii). The expression of circNFIX leads to glioma progression through the increase in ROS. RPN2, which is part of an N-oligosaccharyl transferase complex, is considered oncogenic and a ROS inducer. In gliomas, miR-378e targets RPN2, suppressing its oncogenic functions, for example, by inhibiting ROS production. circNFIX can sponge the miR-378e action, allowing RPN2 activity (as part of the ceRNA network). By doing so, circNFIX alters glucose metabolism, reduces proliferation, and consequently contributes to glioma progression. [99,102]. These data, therefore, suggest a putative oncogenic role for NFIX.

Together, these mechanisms contribute to the regulation of NFIX and ROS levels, which may influence the outcome of cell fate decisions. This is supported by the important role that oxidative stress plays, for example, in proliferation, cell migration and metastasis, and apoptosis [84,86,127,128]. In addition, there is an important link between oxidative stress and glucose metabolism, which has been described not only in cancer [84,86] but also in the context of several other pathologies [129,130,131,132]. In keeping with this notion, studies have suggested that NFIX and circNFIX play an important role in regulating glucose metabolism [99,133]. One example is the above-mentioned activation of RPN2 by circNFIX [99], which has been suggested to promote glycolysis [102]. A recent study has also shown that *Nfix* was downregulated in a mouse model of obesity in response to glucokinase deficiency, a glycolytic enzyme possibly associated with a reduction in oxidative stress levels [133].

The work being reviewed highlights the role of NFIX in cancer, particularly by showing its strict association with increased oxidative stress. Previous studies suggest that NFIX may be a promising prognostic marker [80,94,134], and even though more research is needed to fully understand the relationship between NFIX and ROS in the context of cancer, it is possible that targeting NFIX offers a means of modulating ROS levels in cancer. When designing new therapeutic strategies, it is crucial to consider that targeting NFIX can impact vital cell mechanisms in various cell types, such as hematopoietic, neuronal, or germ cells. Strategies, such as using adeno-associated virus (AAV)-based gene therapy for efficient and tissue/cell-specific delivery of NFIX silencing molecules (including miRNA or lncRNA), could provide a successful approach. Additionally, a growing number of therapeutic approaches are currently being established to increase ROS levels in cancer cells to a point that surpasses the cells’ redox tolerance, triggering an overt oxidative stress response that ultimately may lead to cancer cell death [135]. A better understanding of how NFIX crosstalks with the oxidative stress response may provide yet another application for the modulation of NFIX levels in cancer treatment.

## 5. Conclusions

Over the past few decades, NFIX has primarily been studied in the context of skeletal muscle development and muscle dystrophies, as well as in relation to neuronal and hematopoietic cell differentiation and fate. In this review, we explored the role of NFIX in cancer and its crosstalk with oxidative stress pathways. Given the crucial function of NFIX in cell differentiation during embryonic development, it is possible that a potential link between NFIX, oxidative stress, and cancer cell dedifferentiation might be a pivotal factor in tumor progression. Collectively, this review increases our understanding of the involvement of NFIX in both development and cancer, which is essential for the establishment of targeted cancer therapies.

## Figures and Tables

**Table 1:** Oncogenic and tumor suppressor roles of NFIX.

Putative Oncogenic Gene Fusions		
**Type of cancer**	**Mechanism**	**References**
Breast	*NFIX-MAST1* promotes proliferation.	[77]
	*BSG-NFIX* fusion present in low copy number and with unknown function.	[79]
Skin	*NFIX–PKN1* fusion with unknown function.	[78]
Sarcoma	*NFIX–STAT6* fusion with unknown function.	[75]
**Oncogene**		
**Type of cancer**	**Mechanism**	**References**
Pancreas	ceRNA network: MAFG-AS1 binds to miR-3196 leading to NFIX expression.	[30]
Lung	ceRNA network: SNHG3 binds to miR-1343-3p leading to NFIX expression.	[29]
	NFIX regulates genes involved in proliferation, migration, and invasion (IL6ST, TIMP1 and ITGB1).	[80]
Brain	NFIX upregulates ezrin (EZR) promoting cell migration.	[81]
Prostate	NFIX binds to FOXA1 regulating prostate-specific gene expression.	[82]
**Putative Tumor Suppressor**		
**Type of cancer**	**Mechanism**	**References**
Esophageal	miR-1290 binds to NFIX, decreasing its expression.	[25]
Colorectal	miR-647 and miR-1914 co-target NFIX, decreasing its expression.	[20]
Ovarian	miR-744 reduces NFIX expression, leading to apoptosis.	[22]

## Abbreviations

**Abbreviation**	**Definition**
ADAM10	Disintegrin and metalloproteinase domain-containing protein 10
ATF3	Activating transcription factor 3
BCL2	B-cell leukemia/lymphoma 2
Bcl2l1/BCL-XL	Bcl-2-like protein 1 or B-cell lymphoma-extra large
c-MLP	Thrombopoietin receptor or myeloproliferative leukemia protein
ceRNAs	Competing endogenous RNAs
circRNA	Circular RNA
CKM	Muscle creatine kinase
CYP1A1	Cytochrome P450 1A1
EMX2	Empty spiracles homolog 2
E2A	Immunoglobulin enhancer-binding factors
EBF	Early B cell factor
ENO3	β-enolase
ERK	Extracellular signal-regulated kinases
EZR	Ezrin
GDNF	Glial cell derived neurotrophic factor
IL6ST	Interleukin-6 receptor subunit β
HES	Hairy and enhancer of split-1
HOX	Homeobox genes
*Insc*	Inscuteable
ITGB1	Integrin β-1
JAK/STAT	Janus kinase/signal transducer and activator of transcription
JUNB	AP-1 transcription factor subunit
lncRNA	Long non-coding RNA
MHC	Myosin heavy chain
MAL	Megakaryocytic acute leukemia
MAPK	Mitogen-activated protein kinase
MASH1	Homologue of ASCL1, Achaete-scute homolog 1
MAST1	Microtubule Associated Serine/Threonine Kinase 1
miRNA	microRNA
NFI	Nuclear factor I
NFIX	Nuclear factor I X
NRF2	Nuclear factor erythroid 2-related factor 2
NURF	Nucleosome remodeling factor
PAX6/7	Paired box gene 6/7
PI3K/AKT	Phosphoinositide 3-kinase/protein kinase B
*Pir*	Pirin
PKN1	Protein Kinase N1
RAC1/2	Rac Family Small GTPase 1/2
RhoA	Ras Homolog Family Member A
RPN2	Ribophorin-II
ROCK	Rho associated coiled-coil containing protein kinase 1
ROS	Reactive oxygen species
SRF	Serum response factor
STAT3/6	Signal transducer and activator of transcription 3/6
SOX	SRY-Box Transcription Factor
TGF-β	Transforming growth factor-beta
TIMP1	Metalloproteinase inhibitor 1
